# Effect of Exposure to Smoking in Movies on Young Adult Smoking in New Zealand

**DOI:** 10.1371/journal.pone.0148692

**Published:** 2016-03-09

**Authors:** Philip Gendall, Janet Hoek, Richard Edwards, Stanton Glantz

**Affiliations:** 1 Department of Marketing, University of Otago, Dunedin, New Zealand; 2 Department of Public Health, University of Otago, Wellington, New Zealand; 3 Centre for Tobacco Control Research and Education, University of California San Francisco, San Francisco, California, United States of America; University of Cincinnati College of Medicine, UNITED STATES

## Abstract

**Onscreen Smoking Is a Form of Tobacco Marketing:**

Tobacco advertising has been prohibited in New Zealand since 1990, and the government has set a goal of becoming a smokefree nation by 2025. However, tobacco marketing persists indirectly through smoking in motion pictures, and there is strong evidence that exposure to onscreen smoking causes young people to start smoking. We investigated the relationship between exposure to smoking in movies and youth smoking initiation among New Zealand young adults. Data from an online survey of 419 smokers and non-smokers aged 18 to 25 were used to estimate respondents’ exposure to smoking occurrences in 50 randomly-selected movies from the 423 US top box office movies released between 2008 and 2012. Analyses involved calculating movie smoking exposure (MSE) for each respondent, using logistic regression to analyse the relationship between MSE and current smoking behaviour, and estimating the attributable fraction due to smoking in movies.

**Effect of Smoking in Movies on New Zealand Youth:**

Exposure to smoking occurrences in movies was associated with current smoking status. After allowing for the influence of family, friends and co-workers, age and rebelliousness, respondents’ likelihood of smoking increased by 11% for every 100-incident increase in exposure to smoking incidents, (aOR1.11; p< .05). The estimated attributable fraction due to smoking in movies was 54%; this risk could be substantially reduced by eliminating smoking from movies currently rated as appropriate for youth. We conclude that exposure to smoking in movies remains a potent risk factor associated with smoking among young adults, even in a progressive tobacco control setting such as New Zealand. Harmonising the age of legal tobacco purchase (18) with the age at which it is legal to view smoking in movies would support New Zealand’s smokefree 2025 goal.

## Introduction

The US Surgeon General and other authorities have concluded that tobacco marketing causes young people to start smoking [1–4]. In response to this evidence, and to implement Article 13 of the World Health Organization Framework Convention on Tobacco Control (FCTC), many governments have restricted tobacco promotions, thus limiting young people’s exposure to aspirational smoker identities [5, 6]. Yet, tobacco marketing persists indirectly through onscreen smoking in motion pictures [2, 4, 7–9] and when individuals whom adolescents and young adults admire model smoking, they promote smoking experimentation and uptake [10, 11]. Exposure to smoking in movies fosters perceptions that smoking is normal and enjoyable [12, 13], and leads young people to overestimate smoking prevalence, a misperception that increases smoking initiation [4, 8, 14], while underestimating the risks smoking presents [12, 15, 16].

Evidence of a dose-response relationship between exposure to smoking in movies and smoking prevalence [1, 2, 17–23] led the US Surgeon General to conclude that exposure to onscreen smoking *causes* youth smoking [4]. Depicting smoking in movies thus circumvents FCTC restrictions on tobacco marketing and undermines efforts to reduce smoking prevalence among young people [9]. New Zealand has a particular interest in reducing youth and young adult smoking because the government has banned tobacco advertising and promotion, and set a goal of becoming essentially smokefree by 2025 (defined as smoking prevalence falling below five percent in all population groups) [24]. Many other countries have now set or are considering similar goals [25], making analysis of covert tobacco marketing’s effects on young people a priority.

To test whether residual tobacco marketing in New Zealand affects young people’s smoking behaviour, we followed Song *et al*.’s approach [23] and examined exposure to smoking imagery in movies, the relationship between exposure to smoking in movies and smoking behaviour, and how reductions in exposure to smoking in movies would affect young adults’ risk of smoking initiation. Our aim was to determine what effect, if any, exposure to smoking in movies has in a market where tobacco marketing is otherwise heavily restricted.

## Materials and Methods

### Study Population

Data used were from a survey of 419 respondents aged between 18 and 25, sampled from Research Now’s ‘Valued Opinions’ on-line panel. Research Now has the largest research-only on-line panel in New Zealand, with over 60,000 active members (defined as those who have either updated their panel profile or responded to a survey invitation in the last 12 months). Between 13 and 16 November 2013, participants between 18 and 25 were emailed an invitation directing them to our survey, which was hosted on the Qualtrics online platform.

Though the Valued Opinions panel is broadly representative of the New Zealand population, males (particularly young males) are under-represented and females are over-represented in the panel. To address this gender imbalance, we weighted our data to match the age and gender composition of the 2012 New Zealand population between 18 and 25 [26]; all analyses use these weighted data.

Ethical review and approval was undertaken by a delegated authority from the University of Otago’s Human Ethics Committee. Prior to accessing the online survey, respondents were directed to an Information Sheet setting out the study purpose, intended data use, and their rights as research participants. They were given a researcher’s contact details, should they have any questions, and advised that completion of the survey would be assumed to imply consent.

### Variables

#### Dependent variable

*Smoking Behaviour*: Like Song *et al*. [23], we assessed respondents’ current smoking status by asking smokers: *On the days you smoked during the last 30 days*, *how many cigarettes did you usually smoke each day*? We classified respondents who reported they had smoked at least one cigarette in the last 30 days as current smokers, and all others as non-smokers.

#### Independent variables

*Demographics*: We collected details of respondents’ age, gender, ethnicity, education level, and socio-economic status. Participants were classified either as Māori/Pacific ethnicity while all others (New Zealand European, Chinese, Indian, other Asian or other) were classified as ‘NZ European/Other’. Education was recorded using five categories ranging from no formal qualification to postgraduate qualification, and coded into three groups: school qualification or below; certificate or other qualification below a degree, and Bachelor’s degree or above. We measured socioeconomic status (SES) by asking: *In our society there are groups that tend to be at the top and groups that tend to be at the bottom*. *Below is a scale that runs from top to bottom; 10 being the top and 1 being the bottom*. *Where would you put yourself on this scale*? Participants rated themselves on a scale of one (bottom) to ten (top); this measure has been used in several international social surveys [27].

**Fig 1:**
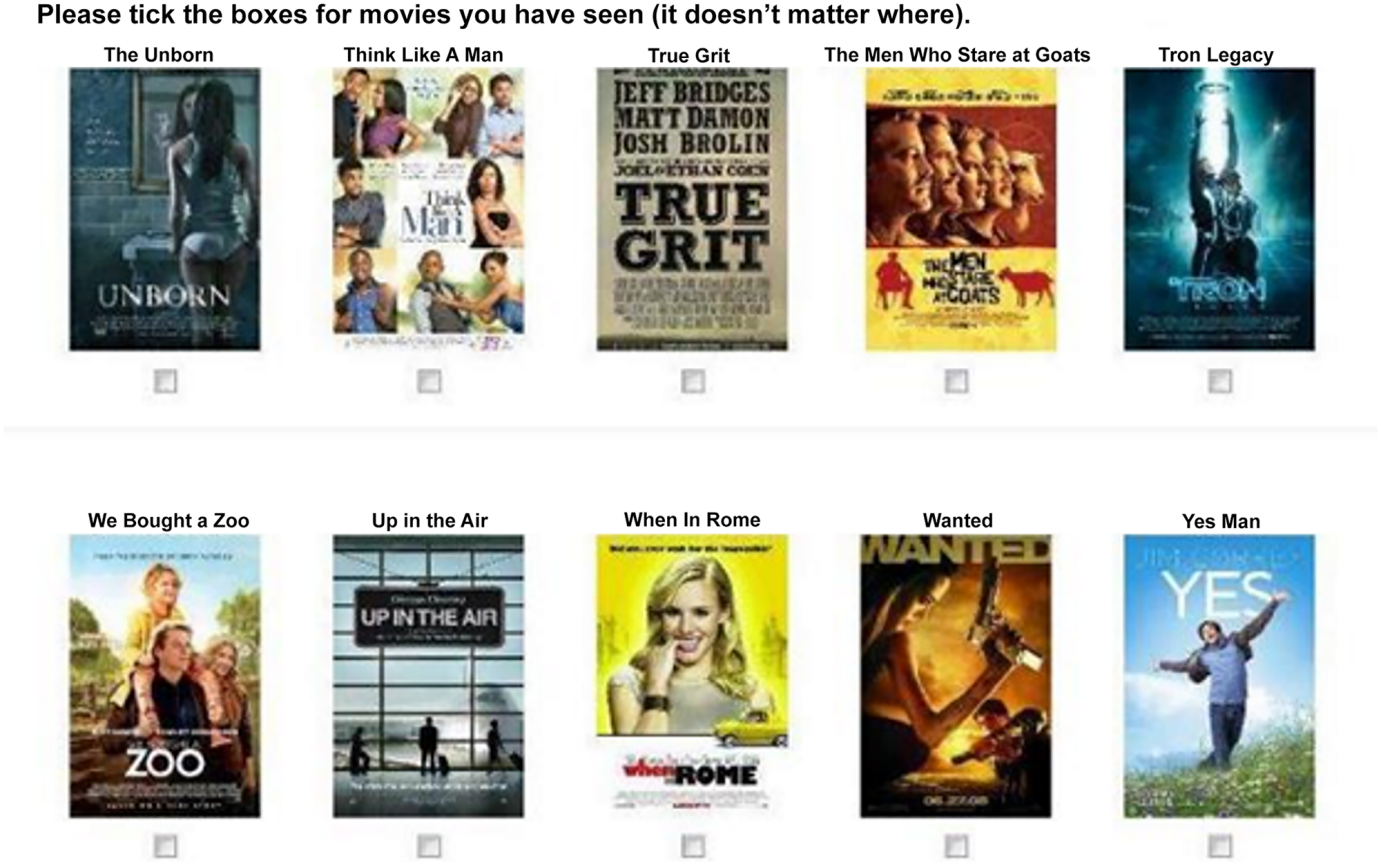
Typical questionnaire screenshot showing movie titles and poster thumbnails.

*Exposure to smoking in movies*: We adopted Sargent *et al*.*’s* approach to estimating exposure to smoking in movies [28, 29], and presented respondents with titles and advertising poster thumbnails of 50 randomly-selected movies (five screens of 10 movies each; see Fig 1) from the 423 US top box office movies released between 2008 and 2012 (see S1 Appendix for details of movies included in the study). Each movie title and thumbnail poster was exposed approximately 60 times and participants indicated which of the movies presented to them they had seen. One ‘dummy’ movie, *Handsome Jack*, served as a control; only four respondents (1%) reported viewing this movie. This low level of erroneous reporting suggests false and random positive reporting did not present serious threats to the data quality.

We obtained details of on-screen occurrences of smoking in each movie from the Dartmouth Media Lab at Geisel School of Medicine, which uses a systematic procedure for identifying smoking occurrences [30]. Of the 423 real movies, 191 (45%) contained smoking scenes; the number of smoking occurrences ranged from 1 to 38, with a median of three per movie. We calculated exposure to smoking in movies using Sargent *et al*.*’s* methodology [21], which summed the smoking depictions in the movies each respondent had seen, divided this figure by the total number of smoking occurrences in the set of 50 movies they viewed, then multiplied this ratio by the total number of smoking occurrences in the 423 movies (1291). This calculation gave each respondent’s movie smoking exposure (MSE).

*Social influences (exposure to smoking in real life)*: We measured social influences by asking participants if their parents (mother, father, other caregivers), siblings (brothers, sisters, cousins), or friends (best friend, other close friend) smoked. If participants answered ‘yes’ for anyone within each group, they were categorised as being influenced by the smoking behaviour of others. The influence of co-workers was measured by a dichotomous variable reflecting whether or not the respondent worked with smokers.

*Personality attributes*: Both sensation seeking and rebelliousness have been strongly associated with smoking among young adults [18, 22]; we measured sensation seeking using two questions used by Morgenstern *et al*. [22] in their study of the effects of smoking in movies on adolescent smoking in Europe (originally developed for use in large-scale surveys by Stephenson *et al*. [31]): *How often do you do dangerous things for fun*? and *How often do you do exciting things*, *even if they are dangerous*? Each question had four possible responses that ranged from ‘Never’ to ‘Often’. Cronbach’s α was 0.86 for the two questions, so we summed the responses and divided by 2.

To assess rebelliousness, we also adopted the approach taken by Morgenstern *et al*. [22] Participants were asked how strongly they agreed that the following statements applied to them: *I believe in following rules* and *I get angry when anyone tells me what to do*; for each question there were four responses ranging from ‘Not at all’ to ‘Very well’. As Cronbach’s alpha was only 0.10, we used the individual ‘rebelliousness’ items rather than the scale constructed from them (though only one item–*I believe in following rules*–was significantly related to current smoking behaviour).

*Movie watching*: Participants were asked two questions to gauge their general movie watching habits: *How often do you watch movies in a cinema*? and *How often do you watch movies on TV*, *on hired video or DVD or from internet download*? The frequency of viewing for these two questions were scored as per Laugesen *et al*. (three or more times a week = 12, once a week = 4, two or three times a month = 2.5, once a month = 1, less than once a month = 0.5 and never = 0) [32]. The scores for both questions were summed and categorised into either fewer than five movies or five or more movies per month (at or below, or above the median number of movies watched per month).

### Statistical Analyses

**Fig 2:**
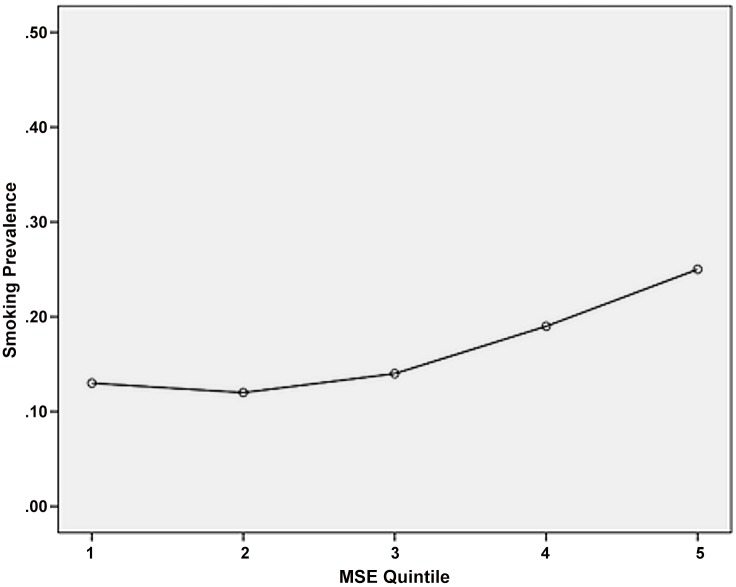
Smoking prevalence vs movie smoking exposure.

We used univariate and multivariate logistic regression analyses to examine the relationship between smoking status and exposure to smoking in movies (MSE) with each one-point increment representing an increase in dose of 100 movie smoking occurrences (see Fig 2). This approach is similar to Sargent *et al*. [21], though we did not ‘Windsorize’ the data by recoding extreme values to those at the second and 98th percentiles to reduce the effect of outliers. Sensitivity analyses with varying degrees of Windsorizing (up to the seventh and 93rd percentiles) yielded only minor differences in the estimated odds ratios, meaning this adjustment would make little difference. All analyses were conducted using SPSS v22.

#### Attributable fraction

Attributable fraction (AF) estimates the reduction in outcome incidence that would be observed if an observed exposure-outcome association is causal and if the population were entirely unexposed compared to its current exposure pattern. AF expresses the proportion of disease incidence (or disease risk) that may be attributed to a specific exposure (in this case, to smoking in movies). We estimated the AF% using the approach first proposed by Bruzzi *et al*. [33], which involved estimating a logistic regression equation with 30-day smoking status as the dependent variable, and behavioural and demographic covariates as independent variables. Using the coefficients from the logistic regression containing all the covariates we estimated the number of smokers in the sample, C_i_. By setting the value of movie smoking exposure for each respondent to zero, we estimated the number of 30-day smokers, assuming no exposure to smoking in movies, C_0_. We then calculated the attributable fraction due to smoking in movies as AF% = (C_i_- C_0_)/C_i_)*100 where: C_i_ = Estimated number of smokers in the population and C_0_ = Estimated number if smoking exposure = 0.

## Results

Young adults aged 18–25 years make up approximately 14% of the population of New Zealand and are slightly over-represented in the Research Now panel, where this group constitutes 20% of the total panel. Because the panel over-represents women, we applied loose gender quotas on recruitment. Survey invitations were emailed to 8397 panel members; of these 556 attempted the survey and 419 completed. Calculating a traditional response rate is not possible, but 75.4% of those who qualified to begin the survey completed it.

Table 1 shows details of the weighted sample’s demographic profile by smoking status. The proportion of smokers in our weighted sample is 16.7%; this compares to smoking prevalence of 16.8% of New Zealanders aged 16 to 25 reported in the 2013 census [26]. Our sample was better educated than the general population of 18 to 35 year olds, had a higher socio-economic status, and had fewer Maori and Pacific peoples; nevertheless smoking prevalence among sample members matched population data for this demographic. Smokers were slightly older, more likely to have friends, family and co-workers who smoke, and were more adventurous and more rebellious than non-smokers. Smokers watched more movies than non-smokers and consequently had more opportunity to be exposed to smoking in movies.

**Table 1:** Weighted sample demographics by smoking status.

Characteristic	Smoking Status^1^		Sig p^2^	Characteristic	Smoking Status^1^		Sig p^2^
	Smoker(n = 70)%	Non-smoker(n = 349)%			Smoker(n = 70)%	Non-smoker(n = 349)%	
**Sex**				**Family or friends smoke**			
Male	15.7	84.3	.331	Yes	24.3	75.7	.000
Female	17.8	82.2		No	5.9	94.1	
**Age**				**Co-workers smoke**			
Mean age	22.1	20.8	.000	Yes	22.2	77.8	.021
**Ethnicity**				No	13.5	86.5	
NZ European/Other	16.9	83.1	.509	**Socio-economic status**			
Maori/Pasifika	15.6	84.4		Mean score on 1–10 scale	4.37	4.33	.853
**Highest education level**				**Movie watching behaviour**			
None/School only	15.3	84.7	.295	< 5 movies/month	13.9	86.1	.041
Sub-degree	23.4	76.6		5+ movies/ month	21.6	78.4	
Degree or higher	17.2	82.8					
**Sensation Seeking**				**Belief in following rules**			
Mean score on 1–4	2.30	2.01	.004	Mean score on 1–4	2.54	2.15	.000
scale				scale			

^1.^ Smoker defined as having smoked at least one cigarette in the last 30 days.

^2.^ Significance of difference between smokers and non-smokers.

Respondents had seen a median of 11 of the 50 movies to which they were exposed. At the film level, viewership rates ranged from 0% (seven films) to 76% (for *The Hunger Games*). MSE ranged from 0 to 1262 exposures. The distribution was highly positively skewed, with a median of 262, and an interquartile range of 105 to 438.

### Movie exposure and current smoking

Exposure to smoking in movies had a significant relationship with respondents’ current smoking behaviour (Table 2 and Fig 2). The unadjusted odds ratio of 1.14 (CI 1.04 to 1.25, p< .05) indicates that the odds of being a smoker increased by a multiplicative factor of 1.14 for every 100 incidents of smoking respondents saw (MSE). When other explanatory variables were entered into the regression equation, the adjusted odds ratio was similar (1.11; CI 1.00 to 1.24, p < .05).

**Table 2:** Predictors of current smoking (in the past 30 days).

Predictor	Unadjusted OR(95% CI)	Adjusted OR(95% CI)^a^
Movie exposure (per 100 exposures)^b^	**1.14 (1.04–1.25)**^c^	**1.11 (1.00–1.24)**
Friends and relatives who smoke^d^	**3.36 (1.65–6.82)**	**2.96 (1.38–6.36)**
Co-workers who smoke^d^	**1.84 (1.10–3.09)**	1.26 (0.71–2.26)
Age	**1.24 (1.11–1.38)**	**1.29 (1.13–1.47)**
Believe in following rules^e^	**2.09 (1.44–3.02)**	**1.87 (1.24–2.81)**
Sensation seeking^f^	**1.59 (1.15–2.20)**	1.37 (0.93–2.00)
Male gender	0.86 (0.51–1.43)	0.85 (0.48–1.53)
Ethnicity^g^	0.89 (0.38–2.07)	0.50 (0.20–1.27)
Highest education level^h^	1.11 (0.82–1.51)	0.74 (0.50–1.09)
Socio-economic Status^i^	1.01 (0.87–1.19)	0.97 (0.82–1.16)

n = 419.

^a^ Adjusted for all other variables. Hosmer and Lemeshow test χ^2^, 8df = 7.19, p = .516. Nagelkerke R^2^ = 0.21.

^b^ For continuous variables, odds ratios indicate incremental increases in odds of being a current smoker for each coded category of the variable.

^c^ Significant(p < .05) odds ratios are bolded.

^d^ Coded categories are: 0 = No; 1 = Yes.

^e^ Coded categories range from 1 = Very well; 2 = Quite well; 3 = A bit; 4 = Not at all; entered as a continuous variable.

^f^ Coded categories ranging from 1 = Low to 4 = High; entered as a continuous variable.

^g^ Coded categories are: 0 = European/Other; 1 = Maori or Pacific Islander.

^h^ Coded categories ranging from1 = None or school qualifications only; 2 = Sub-degree or diploma; 3 = Degree or above; entered as a continuous variable.

^i^ Coded categories ranging from 1 = Low to 10 = High; entered as a continuous variable.

Respondents whose parents, siblings or best friends smoke were nearly three times more likely to smoke than those not exposed to these social influences (Table 2). The likelihood of being a smoker increased significantly with age, and those who were more rebellious (measured by their response to the statement ‘I believe in following rules’ with the scale reversed) were more likely to smoke than those who were less rebellious.

### Attributable Fraction

Using the number of 30-day smokers predicted by the full equation and by the equation with movie smoking exposure set to zero, we estimated the attributable fraction due to smoking in movies at 54% (95% CI 27% to 80%). In New Zealand, over 97% of movies are rated as appropriate for youth (G, PGR, M, R13, R15, or R16); removing smoking incidents from these non-R18-rated (restricted to adults) movies would have virtually the same effect as removing smoking from all movies.

## Discussion

We examined how exposure to smoking in movies affects New Zealand young adults who live in an otherwise heavily restricted tobacco marketing environment, and where the government has a goal to reduce smoking to minimal levels by 2025. Like other countries that have prohibited mass media tobacco marketing, New Zealand’s current film rating policy nevertheless allows adolescents and young adults to be exposed to smoking occurrences in movies. The fact that the odds ratio for the relationship between daily smoking and movie smoking exposure was relatively unaffected by adjusting for other variables suggests exposure to smoking in movies has an independent association with 30-day smoking prevalence in this age group. The relationship between current smoking and movie smoking exposure in our data is consistent with findings from countries with more liberal tobacco marketing environments [7, 17, 21, 23, 28], and suggests tobacco marketing restrictions should include all media, not merely traditional mass media.

Sargent *et al*.*’s* [21] longitudinal study of US adolescents (age 10–14 at entry and 12–16 at the end of the study) found adjusted hazard ratios of 1.49 (95% CI 1.23–1.81) and 1.33 (1.23–1.81) for each additional 500 incidents of MSE in US PG-13 and R rated films, respectively. In a similar study of adolescent never-smokers in six European countries: Germany, Iceland, Italy, The Netherlands, Poland and the United Kingdom, the adjusted relative risk of smoking initiation associated with exposure to each additional 1000 tobacco occurrences in movies was 1.13 (95% CI 1.08–1.17) [34]. The crude relationship between movie smoking exposure and smoking initiation was significant in all countries; after adjustment, the relationship remained significant in all countries except Italy [34]. Our adjusted OR of 1.11 for smoking for 100 incidents of MSE corresponds to an adjusted OR of 1.11^5^ = 1.69 for 500 incidents of MSE, and lies within the 95% confidence intervals for Sargent *et al*.*’s* estimated effect; however, the equivalent for 1000 incidents of MSE is much higher than the Morgenstern *et al* estimate [34].

The relatively high estimate for New Zealand in our study likely reflects the fact that almost all conventional tobacco marketing has been banned in New Zealand, leaving fewer competing factors to stimulate youth and young adults’ smoking. Smoking in movies is thus one of the few marketing channels remaining (apart from cigarette packs, which have 30% pictorial health warnings). Given the power of tobacco marketing to influence young people, it is arguably not surprising that the effect of smoking in movies, independent of other influences, is strong in New Zealand.

Song *et al*. [23] coded movie smoking exposure in quartiles, which they entered in their analyses as a continuous variable. Their estimates of the odds ratios for the effect of movie smoking exposure on current smoking behaviour were OR = 1.30 (95% CI 1.17 to 1.43) and AOR = 1.21 (95% CI 1.05 to 1.38). Our equivalent estimated odds ratios were virtually the same: OR = 1.28 (95% CI 1.02 to 1.53) and AOR = 1.19 (95% CI 1.01 to 1.61). Similarly, Song *et al*.*’s* estimated odds ratio for the top exposure quartile was 2.20 unadjusted, or 1.77 when adjusted for other variables. We found very similar equivalent odds ratios at OR = 2.09 (95% CI 1.03 to 4.23) and 1.66 (95% CI 0.76 to 3.58), suggesting that the effect of smoking in movies on 18 to 25 year olds is similar in the two countries.

The estimated attributable fraction due to smoking in the movies we studied is 54% (95% CI 27% to 80%). This AF estimate lies within the range of attributable fraction estimates reported for the United States and other jurisdictions. A meta-analysis of six US studies of adolescents estimated the pooled attributable fraction due to smoking in movies at 44% (95% CI 34% to 58%) [3]. Though these studies generally involved much younger respondents and used smoking initiation as their dependent variable, our findings are consistent with this earlier work. Consequently, our result adds further weight to the argument that smoking in movies is a significant determinant of the smoking behaviour of young people.

An extensive meta-analysis of the effects of ‘risk-glorifying’ media exposure on risk-taking behaviours, including smoking, also concluded that there was a positive association between exposure to risk behaviours and performance of those behaviours [35]. The study authors concluded that not only did risk glorification in media formats increase risk-taking inclinations and behaviours in the real world (including smoking behaviour), but that the relationship was causal (and in the case of smoking in movies, ‘risk-glorifying’ exposure simply means movie smoking exposure).

Previously, Laugesen *et al*. demonstrated a dose-response relationship between exposure to R-rated movies (an R- rating typically denotes movies rated R16 or R18+) and smoking among New Zealand adolescents [32]. Watching R-rated movies more than once a month doubled the likelihood that a 14 or 15 year old would experiment with smoking. In America, the 2014 US Surgeon General’s Report concluded that youth rates of tobacco use in America would be reduced by 18% if tobacco incidents and impressions in youth-rated films were eliminated (i.e., all future movies with tobacco incidents received a US R rating). The policy equivalent in New Zealand would be to restrict smoking in movies to those with an R18 rating (the age at which it is legal to buy tobacco). Assuming movie producers would remove smoking incidents to avoid an R18 rating and reach a youth audience, this measure could significantly reduce the number of young adults who smoke.

Environmental factors, particularly exposure to smoking by family members or friends, had a strong influence on respondents’ likelihood of smoking. Furthermore, other influences on smoking, such as alcohol use and the link between drinking and smoking, are also potential mechanisms through which movie viewing could the affect smoking behaviour of young people. However, exposure to smoking in movies remained significant and exhibited a dose-response relationship with smoking behaviour, in the presence of other determinants, in line with other reported observations [4, 7, 19, 21]. Unlike other environmental factors, reducing exposure to smoking in movies is readily amenable to policy intervention that reduces exposures delivered in youth-rated films. Public health authorities, including the World Health Organization [9], have called for an upgrading of motion picture rating systems so these include smoking incidents, alongside explicit sex, graphic violence, and offensive language as a factor that merits a restricted rating. This approach has several advantages, including simplicity. It allows film directors the freedom to include smoking in their films (albeit at the cost of the potential audience size) while greatly reducing the attributable risk of smoking initiation among youth who would otherwise be exposed to onscreen smoking [3, 21, 36].

Our findings have some limitations. First, the data are cross-sectional so we cannot determine the direction of causality. Nevertheless, our findings are highly consistent with evidence from longitudinal studies and meta-analyses from around the world [2, 3, 18, 37]. Second, the sample of movies tested did not include any movies not released in America. While a small number of popular movies shown in New Zealand between 2008 and 2012 would not have been in the top 100 shown in the US, not including these titles in the MSE calculation will have had minimal effect. Third, we accounted for only the direct effect of smoking in movies. While the similarity of the unadjusted and adjusted odds ratios suggest this is the main route via which young people are influenced, indirect effects also influence peer smoking and our results may underestimate the total effect of onscreen smoking [23, 38]. Fourth, our sample under-represented Maori and Pasifika and those with less education; thus our results may underestimate the effect of onscreen smoking, given smoking prevalence is higher among these under-represented groups. Finally, an R18 rating would minimise rather than eliminate exposure as some underage youth may (illegally) view R18 movies. Exposure to smoking in R18 movies may thus continue to influence late onset smoking (i.e., among those aged 18 and over), a behaviour pattern increasingly apparent in New Zealand [39, 40].

Governments have recognised the social, health and economic benefits of reducing smoking initiation among young people, even to the extent of setting endgame goals. However, the marketing restrictions enacted leave an important lacuna that tobacco companies have previously exploited, and may still exploit, by negotiating the insertion of tobacco brands and smoking into movies. Indeed, the implementation guidelines for Article 13 of the WHO Framework Convention on Tobacco Control consider smoking in movies a form of media-based tobacco promotion that needs to be addressed [9]. Policy makers could adopt a straightforward response to this problem by harmonising the age at which it is legal to buy tobacco and the age at which it is legal to view smoking in movies. This response would foster more coherent regulation, protect thousands of young people from an addiction that will inevitably compromise their health and longevity, and support realisation of tobacco endgame objectives.

## Supporting Information

S1 Appendix Movies included in study. (DOCX)

S1 Data (SAV)
