# Supplementary material for: Effect of Exposure to Smoking in Movies on Young Adult Smoking in New Zealand
Source: PLoS One. 2016 Mar 9;11(3):e0148692. doi: 10.1371/journal.pone.0148692 (PMC4784919; doi:10.1371/journal.pone.0148692)
Supplement: S1 Appendix — (DOCX) [file pone.0148692.s001.docx]

**APPENDIX: Movies included in study**

| -(500) Days of Summer  -17 Again  -2012  -21 Jump Street  -30 Minutes or Less  -50/50  -9  -Nightmare on Elm Street  -A Perfect Getaway  -A Thousand Words  -A Very Harold & Kumar 3D Christmas  -Abduction  -Adventureland  -African Cats  -Aliens in the Attic  -All About Steve  -Alpha and Omega  -Alvin & The Chipmunks The Squeakquel  -Apollo 18  -Appaloosa  -Armored  -Arthur  -Australia  -Avatar  -Babylon A.D.  -Baby Mama  -Bad Teacher  -Battle: Los Angeles  -Battleship  -Beastly  -Bedtime Stories  -Beverly Hills Chihuahua  -Big Miracle  -Black Swan  -Body of Lies  -Bolt  -Bride Wars  -Bridesmaids  -Brothers  -Burlesque  -Burn After Reading  -Captain America: The First Avenger  -Cars 2  -Changeling  -Charlie St. Cloud  -Chimpanzee  -Chronicle  -Clash of the Titans  -Cloudy with a Chance of Meatballs  -Columbiana  -Conan the Barbarian 35  -Confessions of a Shopaholic  -Contagion  -Contraband  -Cop Out  -Coraline  -Couples Retreat  -Courageous  -Cowboys & Aliens  -Crazy Heart  -Cyrus  -Dance Flick  -Dark Shadows  -Date Night  -Daybreakers  -Dear John  -Death at a Funeral  -Death Race  -Defiance  -Despicable Me  -Devil  -Diary of a Wimpy Kid: Rodrick Rules  -Diary of a Wimpy Kid  -Did You Hear About the Morgans?  -Dinner for Schmucks  -District 9  -Dolphin Tale  -Don't Be Afraid of the Dark  -Drag Me to Hell  -Dream Houce  -Drive Angry 3D  -Drive  -Due Date  -Duplicity  -Eagle Eye  -Easy A  -Eat Pray Love  -Edge of Darkness  -Expelled: No Intelligence Allowed  -Extraordinary Measures  -Fantastic Mr. Fox  -Fast & Furious (2009)  -Fast Five  -Fighting  -Final Destination 5  -Fireproof  -Footloose  -For Colored Girls  -Forgetting Sarah Marshall  -Four Christmases  -Friday the 13th  -Friends with Benefits  -From Paris with Love  -Funny People  -Furry Vengeance  -G-Force  -Gamer  -Get Him to the Greek  -Get Smart  -Ghost Rider: Spirit of Vengeance  -Ghosts of Girlfriends Past  -Handsome Jack  -Going the Distance  -Good Deeds  -Gran Torino  -Green Lantern  -Green Zone  -Grown Ups  -Gulliver's Travels  -Hall Pass  -Halloween II  -Hanna  -Hannah Montana: The Movie  -Happy Feet Two  -Harry Potter & the Deathly Hallows: Part 1  -Harry Potter & the Deathly Hallows: Part 2  -Haywire  -He's Just Not That Into You  -Hellboy 2: The Golden Army  -Hereafter  -High School Musical 3  -Hop  -Horrible Bosses  -Hot Tub Time Machine  -Hotel for Dogs  -How Do You Know  -How to Train Your Dragon  -Hugo  -I Am Number Four  -I Can Do Bad All By Myself  -I Love You, Man | -Imagine That  -Immortals  -In Time  -Inception  -Inglorious Basterds  -Insidious  -Invictus  -Iron Man 2  -Iron Man  -It's Complicated  -Jack and Jill  -Jackass 3D  -Journey 2: The Mysterious Island  -Joyful Noise  -Julie & Julia  -Jumping The Broom  -Just Go With It  -Just Wright  -Kick-Ass  -Killer Elite  -Killers  -Knight and Day  -Knowing  -Kung Fu Panda 2  -Kung Fu Panda  -Lakeview Terrace  -Land of the Lost  -Larry Crowne  -Law Abiding Citizen  -Leap Year  -Leatherheads  -J. Edgar  -Legend of the Guardians: The Owls of Ga'Hoole  -Legion  -Letters to Juliet  -Life As We Know It  -Limitless  -Little Fockers  -Lottery Ticket  -Love & Other Drugs  -Machete  -Madagascar 2  -Made of Honor  -Madea Goes to Jail  -Madea's Big Happy Family  -Madea's Witness Protection  -Magic Mike  -Mamma Mia  -Man on a Ledge  -Marley & Me  -Marmaduke  -Mars Needs Moms  -Max Payne  -Meet Dave  -Megamind  -Midnight In Paris  -Milk  -Mirror Mirror  -Moneyball  -Monte Carlo  -Morning Glory  -Mr. Popper's Penguins  -My Bloody Valentine 3D  -My Sister's Keeper  -My Best Friend's Girl  -New in Town  -New Year's Eve  -Nick & Norah's Infinite Playlist  -Night at the Museum: Battle of the Smithsonian  -Ninja Assassin  -No Strings Attached  -Notorious  -Observe and Report  -Obsessed  -Old Dogs  -One for the Money  -Orphan  -Our Family Wedding  -Our Idiot Brother  -Paranormal Activity 2  -Paranormal Activity 3  -ParaNorman  -Paul Blart Mall Cop  -Paul  -Percy Jackson & The Olympians: The Lightning Thief  -Pineapple Express  -Pirates of the Caribean: On Stranger Tides  -Pitch Perfect  -Planet 51  -Precious Based on the Novel Push by Sapphire  -Predators  -Priest  -Prince of Persia: The Sands of Time  -Project X  -Prom  -Public Enemies  -Push  -Puss in Boots  -007: Quantum of Solace  -Quarantine  -Race to Witch Mountain  -Rango  -Reel Steel  -Red Riding Hood  -Red  -Remember Me  -Repo Men  -Resident Evil 4: Afterlife 3D  -Righteous Kill  -Rio  -Rise of the Planet of the Apes  -Robin Hood  -Rock of Ages  -Safe House  -Salt  -Sanctum  -Savages  -Saw 3D: The Final Chapter  -Saw VI  -Saw V  -Scott Pilgrim vs. The World  -Scream 4  -Season of the Witch  -Secretariat  -Seven Pounds  -Sex and the City 2  -Sex and the City  -Shark Night 3D  -She's Out of My League  -Sherlock Holmes  -Shrek Forever After  -Shutter Island  -Slumdog Millionaire  -Snow White and the Huntsmen  -Something Borrowed  -Soul Surfer  -Source Code  -Splice  -Think Like A Man  -This Is It | -Speed Racer  -Star Wars: The Clone Wars  -State of Play  -Step Up 3D  -Step Brothers  -Sucker Punch  -Super 8  -Surrogates  -Swing Vote  -Taken  -Takers  -Tangled  -That's My Boy  -The Adjustment Bureau  -The Adventures of Tintin  -The Amazing Spider-Man (2012 Reboot)  -The American  -The Back-up Plan  -The Blind Side  -The Book of Eli  -The Bounty Hunter  -The Bourne Legacy  -The Change Up  -The Chronicles of Narnia: The Voyage of the Dawn Treader  -The Crazies  -The Curious Case of Benjamin Button  -The Darkest Hour  -The Day the Earth Stood Still  -The Descendents  -The Devil Inside  -The Dictator  -Spy Kids: All the Time in the World  -Star Trek  -The Dilemma  -The Eagle  -The Expendables  -The Eye  -The Fighter  -The Final Destination (2009)  -The Five-Year Enagagement  -The Fourth Kind  -The Girl with the Dragon Tattoo  -The Green Hornet  -The Hangover Part II  -The Hangover  -The Haunting in Connecticut  -The Help  -The Hunger Games  -The Ides of March  -The Informant!  -The International  -The Invention of Lying  -Gnomeo & Juliet 3D  -The Iron Lady  -The King's Speech  -The Last Airbender 3D  -The Last Excorcism  -The Last House on the Left  -The Last Song  -The Lincoln Lawyer  -The Lorax  -The Losers  -The Lovely Bones  -The Lucky One  -The Mechanic  -The Men Who Stare at Goats  -The Muppets  -The Odd Life of Timothy Green  -The Other Guys  -The Pink Panther 2  -The Princess and the Frog  -The Proposal  -The Roommate  -The Sitter  -The Smurfs  -The Social Network  -The Soloist  -The Sorcerer's Apprententice  -The Spirit  -The Spy Next Door  -The Stepfather  -The Strangers  -The Switch  -The Tale of Despereaux  -The Three Musketeers  -The Time Traveler's Wife  -The Tourist  -The Town  -The Twilight Saga: Breaking Dawn Part 1  -The Ugly Truth  -The Unborn  -The Uninvited  -The Vow  -The Watch  -The Wolfman  -The Woman in Black  -This Means War  -Tower Heist  -Toy Story 3  -Traitor  -Transformers: Dark Side of the Moon  -Transporter 3  -Tron: Legacy  -Tropic Thunder  -Trouble with the Curve  -True Grit  -Underworld: Rise of the Lycans  -Underworld: Awakening  -Unknown  -Unstoppable  -Untraceable  -Up in the Air  -Up  -Valkrie  -Vampires Suck  -Vicky Christina Barcelona  -Wall Street: Money Never Sleeps  -Wanted  -War Horse  -Watchmen  -Water for Elephants  -We Bought a Zoo  -What Happens In Vegas  -What to Expect When You're Expecting  -When In Rome  -Where the Wild Things Are  -Why Did I Get Married Too  -Winnie the Pooh  -Wrath of the Titans  -X-Men Origins: Wolverine  -X-Men: First Class  -Year One  -Yes Man  -Yogi Bear  -You Again  -You Don't Mess With the Zohan  -Your Highness  -Zack & Miri Make A Porno  -Zombieland  -Zookeeper  -Romona and Beezus  -Terminator Salvation  -Thor |
| --- | --- | --- |
